# Repurposing of Zika virus live-attenuated vaccine (ZIKV-LAV) strains as oncolytic viruses targeting human glioblastoma multiforme cells

**DOI:** 10.1186/s12967-024-04930-4

**Published:** 2024-02-02

**Authors:** Carla Bianca Luena Victorio, Wisna Novera, Arun Ganasarajah, Joanne Ong, Melisyaa Thomas, Jonas Wu, Hilary Si Yin Toh, Alfred Xuyang Sun, Eng Eong Ooi, Ann-Marie Chacko

**Affiliations:** 1 Laboratory for Translational and Molecular Imaging, Cancer and Stem Cell Biology Programme, Duke-NUS Medical School, Singapore, Singapore 169857; 2 Laboratory of Human Neural Models, Neuroscience and Behavioural Disorders Programme, Duke-NUS Medical School, Singapore, Singapore 169857; 3 Programme in Emerging Infectious Disease, Duke-NUS Medical School, Singapore, Singapore 169857; 4 Division of Cellular and Molecular Research, National Cancer Centre, Singapore, Singapore 169610

**Keywords:** Zika virus, ZIKV, Live-attenuated vaccine, Oncolytic virus, GBM, Immunogenic cell death, Glioblastoma

## Abstract

Glioblastoma multiforme (GBM) is the most common malignant primary brain cancer affecting the adult population. Median overall survival for GBM patients is poor (15 months), primarily due to high rates of tumour recurrence and the paucity of treatment options. Oncolytic virotherapy is a promising treatment alternative for GBM patients, where engineered viruses selectively infect and eradicate cancer cells by inducing cell lysis and eliciting robust anti-tumour immune response. In this study, we evaluated the oncolytic potency of live-attenuated vaccine strains of Zika virus (ZIKV-LAV) against human GBM cells in vitro. Our findings revealed that Axl and integrin α_v_β_5_ function as cellular receptors mediating ZIKV-LAV infection in GBM cells. ZIKV-LAV strains productively infected and lysed human GBM cells but not primary endothelia and terminally differentiated neurons. Upon infection, ZIKV-LAV mediated GBM cell death via apoptosis and pyroptosis. This is the first in-depth molecular dissection of how oncolytic ZIKV infects and induces death in tumour cells.

**Supplementary Information:**

The online version contains supplementary material available at 10.1186/s12967-024-04930-4.

## Introduction

Glioblastoma multiforme (GBM) is the most common malignant primary brain cancer, with > 300,000 patients diagnosed annually worldwide [1–3]. Therapeutic options are limited, resulting in poor median overall survival (m_OS_ = 15 months) in patients despite an aggressive standard of care regime [4, 5]. GBM-directed therapies delivered systemically must primarily be able to penetrate the blood–brain barrier (BBB) and blood–brain tumour barrier (BBTB) for optimal drug delivery [6, 7]. Moreover, tumour recurrence mediated by glioma stem cells (GSCs) refractory to chemical and radiation therapy remains a major challenge in the absence of therapies that effectively eradicate these cells [8–10].

Oncolytic virotherapy, a form of treatment where engineered viruses infect and directly kill tumour cells, is a promising alternative that could potentially address the therapeutic challenges mentioned above. The engineered viruses mediate tumour cell death by (1) hijacking cellular metabolic resources for production of virus progeny, and (2) eliciting durable anti-tumoural immune responses [11, 12]. Several oncolytic viruses (OV) from diverse groups of virus families have been evaluated in preclinical and clinical trials against GBM. Some of these OVs have prolonged the survival of about a third of patients by up to 24 months [13–15]. Further, a third-generation herpes simplex virus-1 (HSV-1), Teserpaturev/ΔG47 (Delytact^™^) was recently approved in Japan for the treatment of malignant glioma [16].

Zika virus (ZIKV), a member of the flavivirus virus family, is one such virus candidate that is in the stage of early experimental development. ZIKV naturally infects neuroprogenitor cells (NPCs) in developing mouse and human foetuses as well as in the adult hippocampi [17–22] and causes global neuroinflammation [23, 24]. Wild type ZIKV clinical isolates were reported to infect and lyse human GSCs in vitro and in vivo [25–27], while mouse-adapted and modified ZIKV strains were reported to inhibit tumour growth in syngeneic and xenograft orthotopic GBM models using both immunocompetent and immunocompromised mice [25, 28–31]. Recently, clinical ZIKV strains have also been employed in the experimental treatment of spontaneous brain tumours in dogs [32]. Though ZIKV treatment inhibited tumour growth and prolonged survival in experimental mouse GBM models, the off-target effects of ZIKV infection in non-tumour tissues have not been thoroughly studied. ZIKV has a wide tissue tropism, including the gonads, eyes, and brain [25, 28], which could pose safety concerns during its use as a therapeutic agent. Thus, the potential of attenuated ZIKV strains that exhibit reduced infectivity in normal tissues—particularly the brain and gonads— in treating syngeneic and xenograft mouse GBM tumours has been studied [29, 31].

ZIKV can be attenuated by various methods, such as deleting genome regions critical for virulence [29, 33] and recoding the genome CpG content [34]. However, attenuation may affect the potency of ZIKV as a therapeutic agent. Specifically, mutations that reduce the replicative fitness of ZIKV may render it safe but may also limit its ability to spread within the tumour mass. As an alternative, we explored the potential of Zika live-attenuated vaccine (ZIKV-LAV) strains that grow rapidly and activate type-I interferon response early in the course of infection. These strains display attenuated infection and dissemination in primary human monocytes [35]. Moreover, the infection of A129 (interferon-α,β receptor knockout) mice with these ZIKV-LAV strains, known as DN-1 and DN-2, did not result in significant disease; the strains exhibited reduced replication in the brains and gonads relative to wild-type ZIKV [35]. In this study, we examined whether DN-1 and DN-2 could selectively infect cancer cells while sparing non-cancer cells from infection—a highly desirable phenotype for OVs. We also tease out the cell death pathways involved in tumour cell killing and identify the cellular receptors used by DN-1 and DN-2 to infect human GBM cells. Our findings will lend valuable insights into the mechanisms employed by oncolytic ZIKV-LAV strains to selectively target and kill human GBM cells in vitro.

## Results

### ZIKV-LAV (Zika virus live-attenuated vaccine) strains productively infect and lyse human GBM cells

**Fig. 1:**
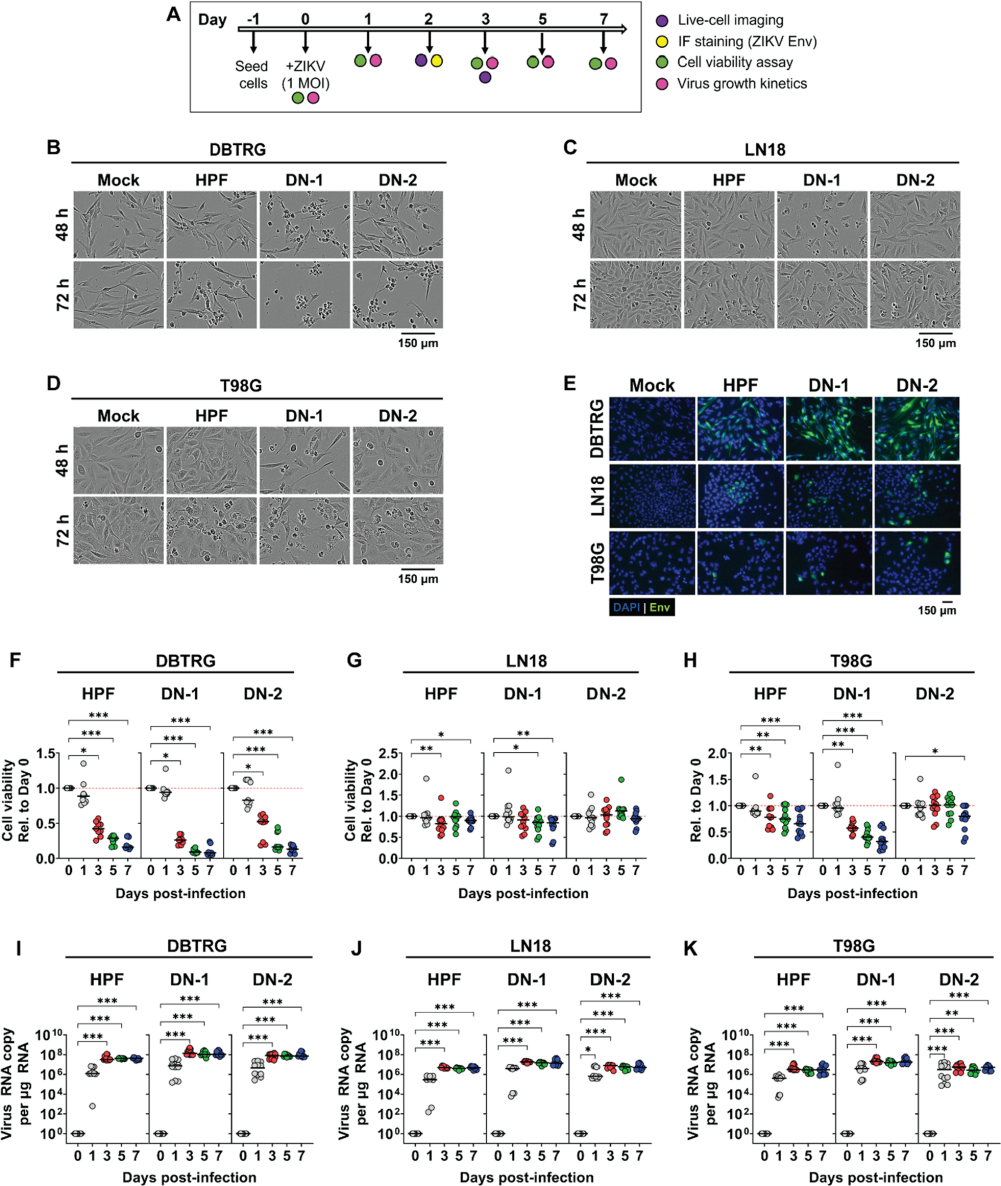
ZIKV-LAV strains productively infect human GBM multiforme (GBM) cell lines. **A** Timeline of in vitro infection experiments. Cells were inoculated with either ZIKV-LAV strains, DN-1 and DN-2, or parent strain HPF, at 1 plaque-forming unit (pfu) per cell. Infected cells were observed for 7 days. **B**–**D** Representative live-cell images of human GBM cell lines **B** DBTRG, **C** LN18, and **D** T98G at 48 h and 72 h post-infection. **E** Representative fluorescence images of infected cells probed for the expression of ZIKV envelope (Env) protein. **F**–**H** Kinetics of cell death in **F** DBTRG, **G** LN18, and **H** T98G cells over 7 days after virus infection. **I**–**K** Virus growth kinetics following infection in **I** DBTRG, **J** LN18, and **K** T98G cells. Data are presented as individual points. Horizontal bars represent medians. Non-parametric Kruskal–Wallis test with Dunn’s post-hoc correction was used to compare groups. *p*-values are shown accordingly: *p < 0.05. **p < 0.005, ***p < 0.001

Various assays were performed to confirm ZIKV-LAV infection in three human GBM cell lines—DBTRG, LN18, and T98G (Fig. 1A). ZIKV-LAV infection led to significant cell death (Fig. 1B–D) and the production of whole virus in infected cells, as confirmed by live-cell imaging and immunofluorescence (IF) staining for ZIKV envelope (Env) protein (Fig. 1E), respectively. ZIKV-LAV infection reduced cell viability over time (Fig. 1F–H), and the effect was more prominent in DBTRG and T98G cells compared to LN18 cells. Cell death was more pronounced in DBTRG and T98G cells that were infected with the ZIKV-LAV DN-1 strain than with the parental wild type ZIKV HPF strain, and this was most obvious on days 3–7 post-infection (Fig. 1B, D, F–H). Despite differences in the cell death phenotype induced by the two ZIKV strains, the viral replication kinetics were comparable across HPF, DN-1, and DN-2 strains, peaking on day 3 post-infection in all the three cell lines evaluated (Fig. 1I–K).

**Fig. 2:**
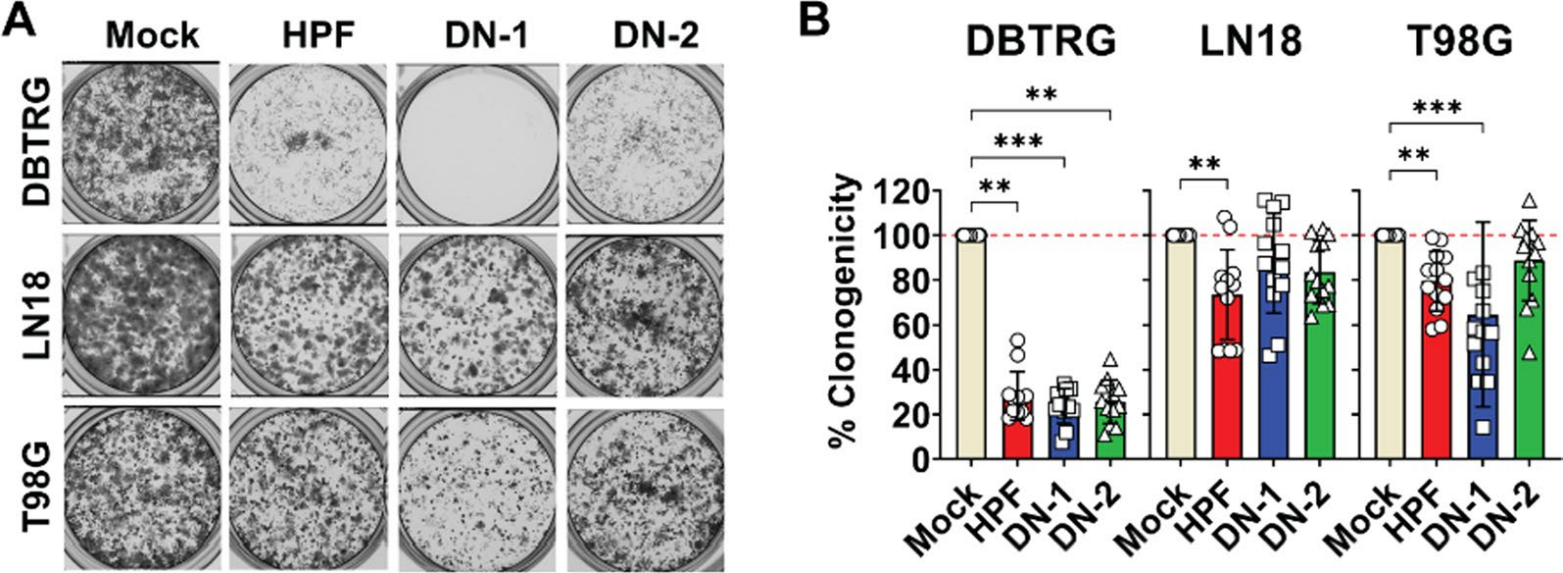
ZIKV-LAV infection inhibits clonogenic reproduction of human GBM cells. Human GBM cells (DBTRG, LN18, and T98G) were inoculated with 1 pfu per cell of either ZIKV-LAV strains (DN-1 and DN-2) or parent HPF strain. Cells were trypsinized and re-seeded at 5,000 cells per well on day 5 post-infection. **A** Representative clonogenicity plates of infected cells. **B** Quantification of clonogenicity data, which are presented as mean ± SEM. Non-parametric Kruskal–Wallis test with Dunn’s post-hoc correction was used to compare groups. *p*-values are shown accordingly: *p < 0.05. **p < 0.005, ***p < 0.001

Furthermore, the clonogenic self-renewal of the three cell lines were differentially altered following 5 days of infection with the various ZIKV-LAV strains (Fig. 2A). For instance, infection with the HPF, DN-1, and DN-2 strains reduced DBTRG clonogenicity by 71.72 ± 10.89%, 76. 02 ± 7.77%, and 74.33 ± 9.76%, respectively (Fig. 2B). Meanwhile, only the HPF and DN-1 strains inhibited T98G cell clonogenicity by 27.52 ± 26.65% (*p* = 0.006) and 41.37 ± 46.58% (*p* < 0.001), respectively. (Fig. 2B). In contrast, only the HPF strain mildly inhibited LN18 cell clonogenicity by 27.52 ± 26.65% (*p* = 0.009), whereas the ZIKV-LAV strains did not affect the clonogenicity of this cell line (Fig. 2B). Similar reduction in cancer cell clonogenicity was observed in cells following only 2 days of ZIKV-LAV infection (Additional file 1: Fig. S1). These results demonstrate that ZIKV-LAV infection in human GBM cells not only causes acute cell death but additionally impacts the clonogenic self-renewal of surviving infected cells. These phenomena contribute to reduced tumour cell growth in vitro following ZIKV-LAV infection.

### ZIKV-LAV does not productively infect non-tumour and normal human cells

**Fig. 3:**
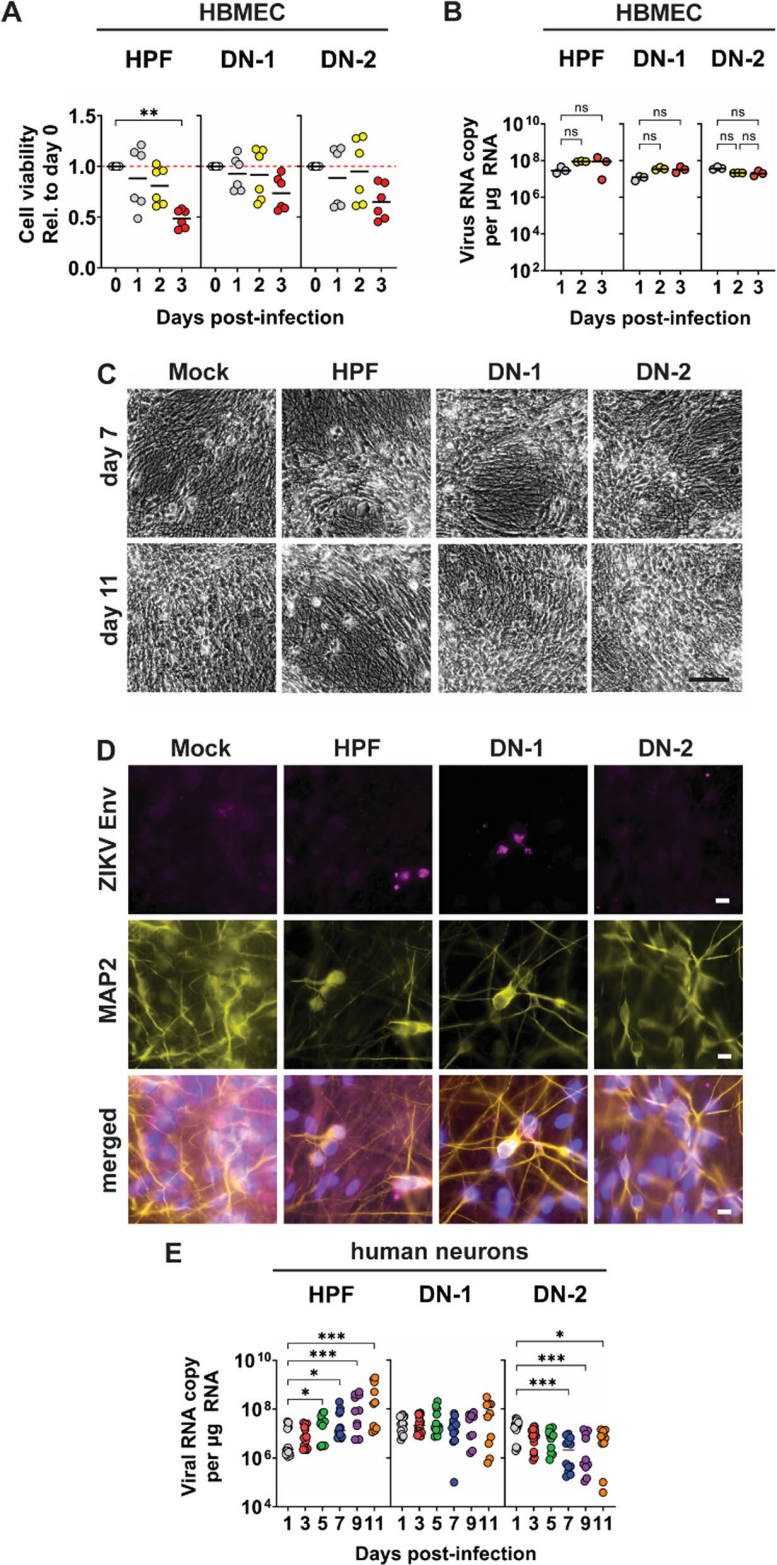
ZIKV-LAV strains exhibit limited infection in normal brain cells. **A**–**B** ZIKV-LAV infection of human brain microvascular endothelia (HBMEC) cells over 3 days, evaluated by measuring changes in cell viability **A** and viral copies **B** detected in infected cells. **C**–**E** ZIKV-LAV infection of cultured human neurons over 11 days. Representative **C** live-cell images of cultured human neurons at day 7 and day 11 post-infection; and **D** immunofluorescence images of cultured human neurons at day 11 post-infection. Infected human neurons were probed for the expression of virus envelope protein (Env) and microtubule-associated protein 2 (MAP2). **E** Viral copies detected from the supernatant of human neuronal culture at 11 days post-infection with ZIKV-LAV. Data are presented as mean ± SEM. Non-parametric Kruskal–Wallis test with Dunn’s post-hoc correction was used to compare groups. *p*-values are shown accordingly: *p < 0.05. **p < 0.005, ***p < 0.001. Scale bar = 250 µm

The infection of HBMEC cells with ZIKV-LAV did not induce cell death (Fig. 3A) or lead to viral replication (Fig. 3B). In contrast, infection of these cells with the parental wild type HPF strain induced significant cell death by day 3 post-infection (Fig. 3A). Consistent with the above findings, ZIKV-LAV also did not infect HUVEC cells, (Additional file 1: Fig. S2), suggesting the limited capacity of these strains to infect non-cancer-derived human cells. Furthermore, ZIKV-LAV did not induce cell death or changes in cytopathology in terminally-differentiated neurons derived from human induced pluripotent stem cells (hIPSCs) (Fig. 3C). This was further confirmed by immunofluorescence (IF) staining for ZIKV envelope (Env) protein (Fig. 3D). Importantly, both ZIKV-LAV strains did not replicate in terminally-differentiated neurons, while the parent HPF viral replication peaked at day 11 post-infection (Fig. 3E). Altogether, our findings confirm that ZIKV-LAV can enter and express viral proteins in cultured human neurons, but their proliferation within these cells is impaired.

### ZIKV-LAV infection induces lytic and non-lytic forms of cell death in human GBM cells

**Fig. 4:**
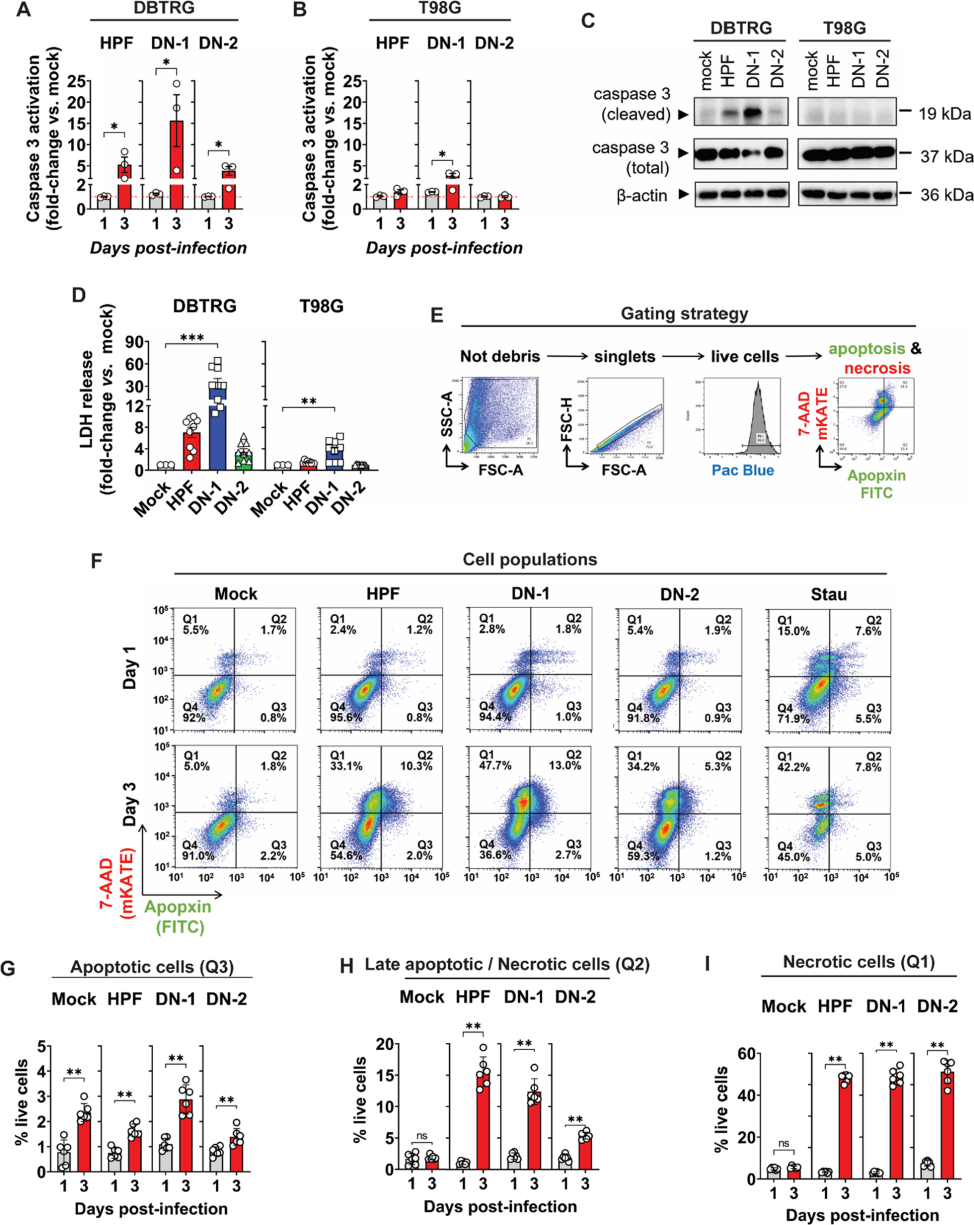
ZIKV-LAV infection induces apoptotic and necrotic cell death in human GBM cells. **A**–**B** Caspase-3 activation for apoptosis in virus-infected **A** DBTRG and **B** T98G cells detected by luminescence assay on days 1 and 3 post-infection. **C** Western blot detection of cleaved caspase-3 in infected cell lysates at day 2 post-infection. **D** Lactose Dehydrogenase (LDH) release by necrotic cells at day 3 post-infection. **E** Gating strategy for the simultaneous detection of apoptotic and necrotic DBTRG cells by flow cytometry. **F** Representative scatter dot-plots of DBTRG cells during early (day 1) and late (day 3) stages of infection with ZIKV-LAV. Cells were co-stained and detected with apopxin-FITC and 7-AAD-mKATE. **G**–**I** Quantification of the fraction of **G** apoptotic, **H** late apoptotic/necrotic, and **I** necrotic DBTRG cells by flow cytometry during late stage of infection with ZIKV-LAV. Values are presented as % of live cells. Data are presented as mean ± SEM. Non-parametric Mann–Whitney test or Kruskal–Wallis test with Dunn’s post-hoc correction was used to compare groups. *p*-values are shown accordingly: *p < 0.05. **p < 0.005, ***p < 0.001

Luminescence assays were performed to detect Caspase-3/7 proteolytic cleavage in DBTRG and T98G cells infected with ZIKV-LAV (Fig. 4A, B); western blots were used to confirm cleaved caspase-3 in DBTRG cells (Fig. 4C). In DBTRG cells, DN-1 infection induced more caspase-3/7 activation (Fig. 4A) and caspase-3 cleavage (Fig. 4C) than HPF infection. Similarly, in T98G cells, only DN-1 infection induced caspase-3/7 activation (Fig. 4B), although caspase-3 cleavage could not be detected by Western blot (Fig. 4C). In addition, in both DBTRG and T98G cells, DN-1 infection led to increased release of lactose dehydrogenase (LDH) into the supernatant compared to mock-infected controls (Fig. 4D). These findings confirm that ZIKV-LAV strains induce both apoptotic (non-lytic) and lytic cell death in human GBM cells. Lytic and non-lytic cell death in these cells were simultaneously monitored and measured by flow cytometry (Fig. 4E). Viral infection led to increased population of DBTRG cells labelled with apopxin (apoptotic) and 7-AAD (lytic) cell markers—similar to the profile of cells treated with the apoptosis-inducing drug staurosporine (Stau) (Fig. 4F). Increased population of apoptotic DBTRG cells (Fig. 4G, H) was seen following infection with either HPF or ZIKV-LAV. However, majority of the infected cells were undergoing lytic or necrotic cell death (Fig. 4H, I). These trends are comparable to findings in T98G cells infected with HPF and ZIKV-LAV (*data not shown*), confirming that cell lysis is the predominant form of cell death induced by ZIKV-LAV in human GBM cells.

### ZIKV-LAV infection leads to pyroptosis in human GBM cells

**Fig. 5:**
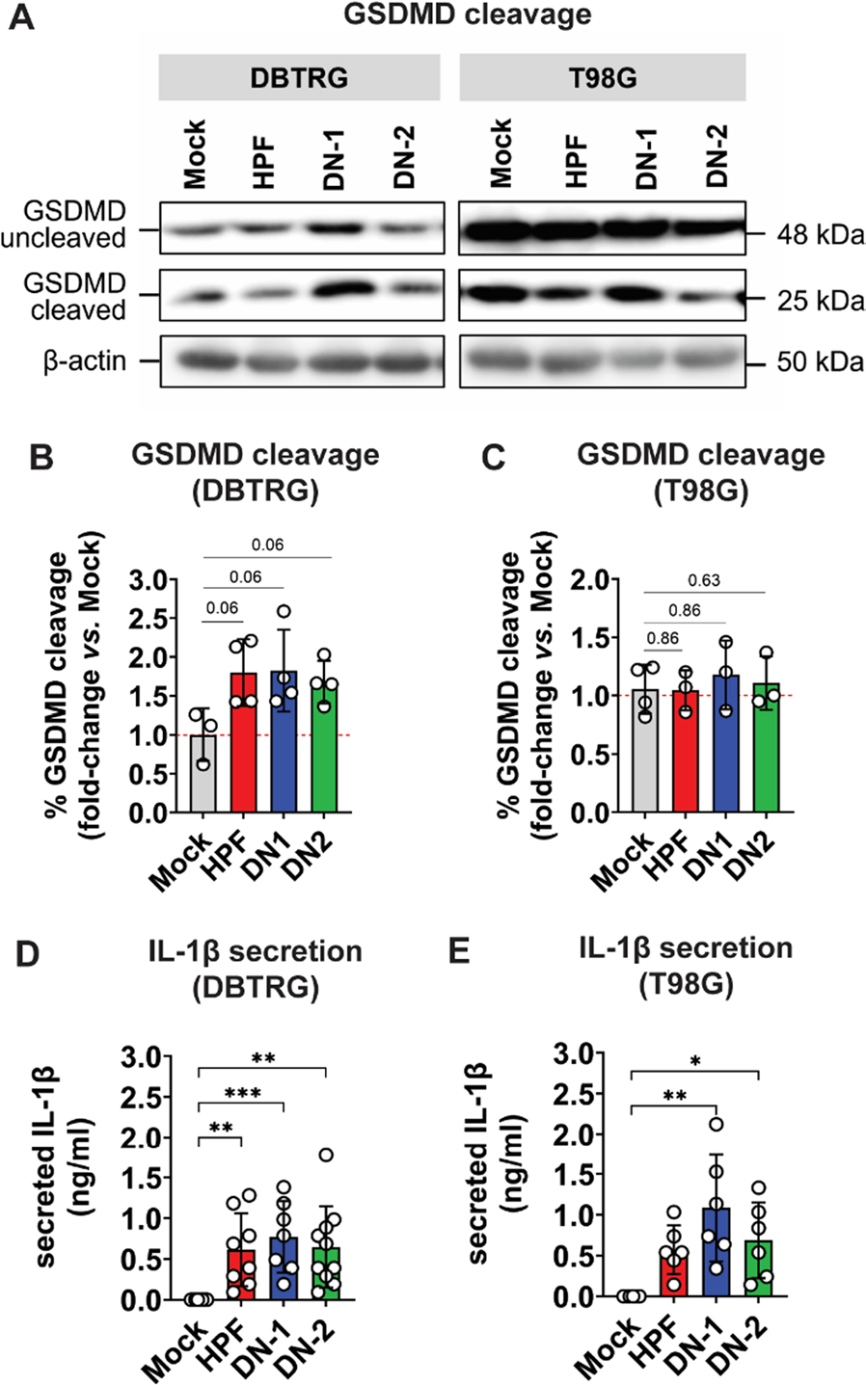
Pyroptotic cell death induced by ZIKV-LAV infection in human GBM cells. **A** Western blot detection of gasdermin D (*GSDMD*) cleavage in infected DBTRG and T98G cell lysates at day 5 post-infection. **B**–**C** Quantification of GSDMD cleavage from western blot data normalized to β-actin expression. Data are shown as fold-change in percentage of (%) GSDMD cleavage relative to mock-infected cells. **D**–**E** Luminescence-based detection of IL-1β secreted by infected cells. Data are shown as mean ± SD. Means were compared by Kruskal–Wallis test with Dun’s post-hoc correction. *p < 0.05; **p < 0.005; ***p < 0.001

DBTRG and T98G cells infected with ZIKV exhibited gasdermin-D (GSDMD) cleavage, as detected by Western blot at late stage (day 5) of infection (Fig. 5A). Only DBTRG cells exhibited a trend of increasing GSDMD cleavage following infection relative to mock-infected cells, but these differences were not statistically significant (*p* < 0.10) (Fig. 5B). In contrast, T98G cells exhibited GSDMD cleavage at a level comparable to mock-infected cells (Fig. 5C). Furthermore, pyroptosis in virus-infected human GBM cells was confirmed by measuring secreted IL-1β in culture supernatants with luminescent ELISA. While infections with both HPF and ZIKV-LAV strains led to elevated IL-1β secretion in DBTRG cells (Fig. 5D), only ZIKV-LAV infection resulted in comparable increase in IL-1β secretion in T98G cells (Fig. 5E). These findings indicate that pyroptosis is one of the lytic forms of cell death induced by ZIKV-LAV infection in human GBM cells.

### ZIKV-LAV use Axl and integrin α_v_β_5_ as receptors to infect human GBM cells

**Fig. 6:**
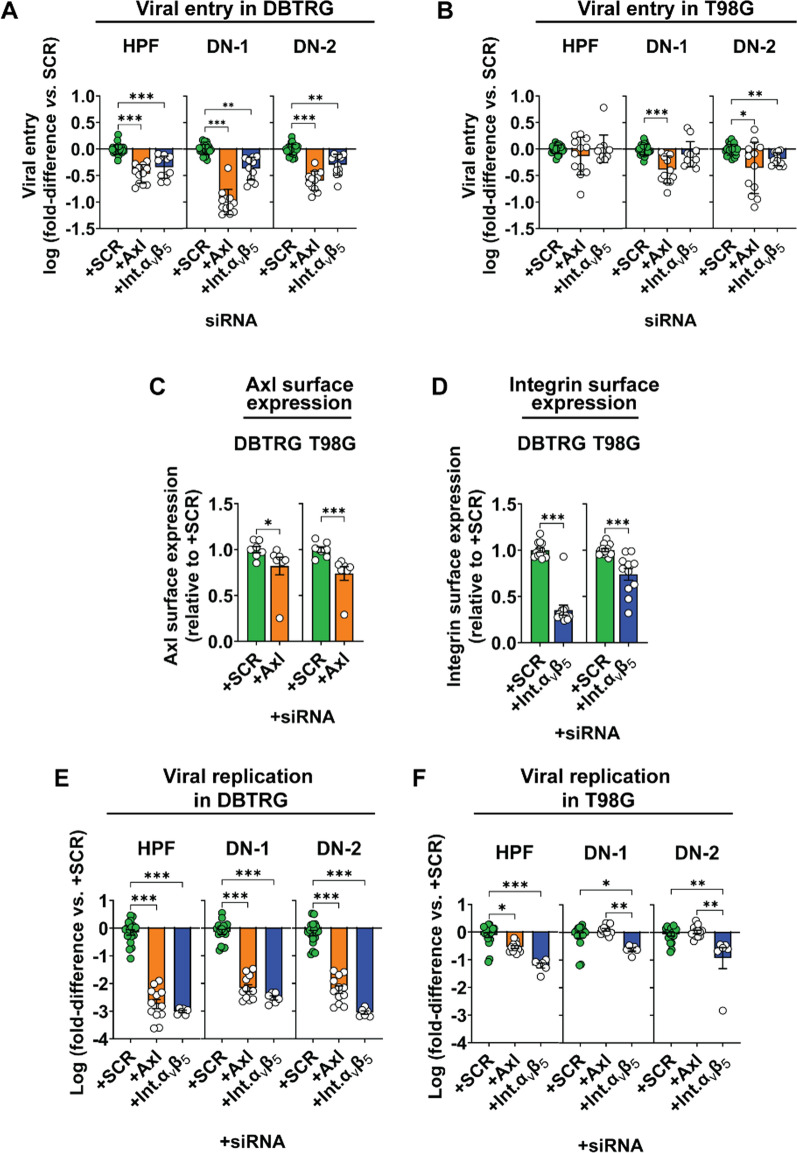
ZIKV-LAV entry in human GBM cells is mediated by Axl and integrin α_v_β_5_. Evaluation of the effect of siRNA-mediated knockdown of either Axl or integrin α_v_β_5_ gene on **A**–**B** viral entry in DBTRG (**A**) and T98G (**B**) cells; **C**–**D** protein expression of Axl (**C**) and integrin α_v_β_5_ (**D**) on the cell surface; and **E**–**F** intracellular viral replication in DBTRG (**E**) and T98G (**F**) cells. *SCR*, scrambled siRNA. Int.α_v_β_5_, integrin α_v_β_5._ Data are presented as mean ± SEM. Non-parametric Mann–Whitney test or Kruskal–Wallis test with Dunn’s post-hoc correction was used to compare groups. *p*-values are shown accordingly: *p < 0.05. **p < 0.005, ***p < 0.001

siRNA-mediated knockdown of receptor tyrosine kinase Axl and integrin α_v_β_5_ expression inhibited entry of ZIKV into DBTRG cells assayed within 1.5 h post-incubation with virus (Fig. 6A). However, similar perturbations of receptor expression in T98G cells affected the entry of only ZIKV-LAV strains (Fig. 6B). We confirmed that siRNA-mediated gene knockdown resulted in reduced cell-surface expression of both Axl (Fig. 6C) and integrin α_v_β_5_ (Fig. 6D) in both cell lines. The data were consistent with the reduction, but not complete knockdown, of gene expression observed in qPCR studies (Additional file 1: Fig. S3). We observed 70–80% and 40–50% siRNA-mediated knockdown of Axl and integrin α_v_β_5_, respectively. Moreover, siRNA-mediated knockdown of both Axl and integrin α_v_β_5_ receptors also reduced viral replication of HPF and ZIKV-LAV in DBTRG cells (Fig. 6E). However, only the knockdown of integrin α_v_β_5_ reduced HPF and ZIKV-LAV replication in T98G cells. These results indicate that Axl and integrin α_v_β_5_ are entry receptors for HPF and ZIKV-LAV to infect DBTRG cells. However, the effect of siRNA knockdown on HPF and ZIKV-LAV infection of T98G cells is less clear. Axl knockdown in T98G cells affected ZIKV-LAV cellular entry but not viral replication; in contrast, integrin α_v_β_5_ knockdown affected intracellular replication without impacting cellular entry of the virus.

## Discussion

The Zika virus live-attenuated vaccine (ZIKV-LAV) strains, particularly DN-1, exhibited potent oncolytic activity in human glioblastoma multiforme (GBM) cells without productively infecting non-cancer cells, such as terminally differentiated human neurons and primary endothelia [35]. The production of ZIKV-LAV in different GBM cells peaked at day 3 post-infection, showing viral growth kinetics comparable to the parent wild-type strain. This suggests ZIKV-LAV undergoes sufficient viral replication in human GBM cells to produce more virus progeny that could effectively infect neighbouring cancer cells. In addition, ZIKV-LAV replication was inversely related to cell viability and clonogenic potential of infected DBTRG and T98G cells, which demonstrates that transient ZIKV-LAV replication in these cancer cells was sufficient to inhibit the uncontrolled cell division in cancer cells and induce cancer cell death. Of the three human GBM cells evaluated, DBTRG was the most permissive to ZIKV-LAV infection, while LN18 was the least permissive. This differential infectivity of various human GBM cells to ZIKV-LAV is expected as it mirrors the observed intra-tumoural heterogeneity within the same patient and inter-tumoural heterogeneity across different GBM patients [36, 37]. These differences maybe be driven by intrinsic expression level of host cellular factors such as Axl and integrin α_v_β_5_ receptors required for ZIKV-LAV infection and replication or evasion of host immune response [38–40].”

ZIKV-LAV infection in non-cancer cells was evaluated as proxy for cancer selectivity of viral infection. Consistent with prior studies, the parent clinical isolate HPF naturally infected HUVEC [41, 42] but ZIKV-LAV strains did not. HBMEC cells were also permissive to both ZIKV HPF and ZIKV-LAV infection. However, while HPF infection induced lytic death in these cells, ZIKV-LAV infection had no effect. The difference in ZIKV-LAV infectivity between HUVEC and HBMEC suggest that brain-derived endothelia may be inherently more susceptible to ZIKV-LAV infection than those derived from the foetal umbilical cord but for reasons that are not fully clear. HBMEC was shown to be persistently infected with ZIKV and could be one of the pathways by which the virus gets transported to the brain [43]. Importantly, the absence of DN-1 and DN-2 infection in terminally-differentiated neurons—at least in vitro—suggests that though ZIKV-LAV strains may spillover from HBMEC to neighbouring neurons, likely through endothelial transcytosis reported for wild-type ZIKV [43, 44], infection in normal neurons is unlikely to occur. When taken as a proxy for cancer cell specificity, the reduced infectivity of ZIKV-LAV on non-cancer cells—a consequence of virus attenuation—provides a therapy safety margin and demonstrates that ZIKV-LAV exhibit potent cancer-selective oncolytic activity in vitro*.*

The tumour cell death pathway induced by ZIKV-LAV infection dictates the potency of anti-tumour immune response elicited by virus infection. ZIKV-LAV infection in human GBM cells leads to both apoptotic and lytic cell death pathways—particularly pyroptosis—during late infection. Non-apoptotic, lytic cell death leads to the release of tumour-associated neo-antigens and cytokines. This immunogenic form of cell death attracts host innate and adaptive immune cell components [12, 13] and induces both local and systemic anti-tumour immunity [45]. Similarly, ZIKV infection of placenta cells [46] and macrophages [47] was reported to induce pyroptosis in vitro*,* whereas ZIKV infection of human astrocytes [48] was reported to cause necroptosis, another form of lytic cell death. Human bone osteosarcoma HOS cells and human lung adenocarcinoma A549 cells infected with oncolytic adenovirus and vaccinia viruses are known to exhibit pyroptotic and necroptotic cell death in vitro [49]. Co-culture of the virus-infected HOS or A549 cells undergoing pyroptosis and necroptosis with PBMCs and subsequent in vitro functional immunological assays in PBMCs revealed increased dendritic cell phagocytosis of OV-infected cancer cells and increased T cell maturation [49]. We were unable to detect any evidence of necroptosis in ZIKV-infected human GBM cells. While total MLKL (mixed lineage kinase domain-like) protein could be probed by Western blot assays, MLKL phosphorylation was undetectable (*data not shown*). Further studies are needed to determine the contribution of other forms of lytic cell death—including necroptosis—in human GBM cell death induced by ZIKV-LAV infection. Although ZIKV-LAV infection activates both humoral and cellular immunity in mice not bearing tumours [50], whether ZIKV-LAV infection of the GBM tumour induces immunogenic cell death needs to be demonstrated in a mouse GBM treatment model. It will be interesting to see whether pyroptotic and necroptotic GBM cell death induced by ZIKV-LAV modulates tumour immune microenvironment in vivo as reported recently [51]*.*

ZIKV-LAV strains infect human GBM cells via Axl and integrin α_v_β_5_ receptors. Axl is a tyrosine kinase receptor found on cell membranes and, according to the Human Protein Atlas, is highly expressed in various tissues, including muscles, bladder, urinary tract, gonads, gastrointestinal tract, and lungs [52]. Axl has been determined as a ZIKV entry receptor for the infection of endothelial [53, 54] and glial cells [55]. According to TCGA (The Cancer Genome Atlas) project TCGA-GBM-L4 v.4.2, no difference in Axl protein expression is noted in human GBM patients with long *vs.* short median survival (Cox regression analysis *p* = 0.54). However, Axl expression delineates the different GBM tumour subtypes (*p* = 0.005)—with highest and lowest expression in neural and proneural subtypes, respectively [56, 57]. Thus, ZIKV-LAV treatment, as a form of oncolytic virotherapy, would be most effective against GBM tumours of the neural subtype. Beyond human GBM, Axl is also overexpressed in various haematological and solid cancers, including lung, breast, prostate, and ovarian cancers [58, 59], thus raising the possibility of employing Axl-mediated ZIKV-LAV infection as a therapeutic option against these patient groups.

On the other hand, integrin α_v_β_5_ is a dimer comprised of subunits α_v_ and β_5_. The β_5_ subunit is constitutively expressed in most tissues, while the α_v_ subunit is highly expressed in the brain, endocrine tissues, kidneys, urinary bladder, gonads, bone marrow and lymphoid tissues [52]. While Integrin α_v_β_5_ has been identified as a ZIKV receptor for the infection of glioma stem cells (GSCs) [60] and neuroprogenitor cells (NPC) [61], its role in cancers and in differentiating tumour types that may benefit from ZIKV-LAV treatment is not well known. An earlier study using immunohistochemical staining of tumour tissue microarrays suggested that integrin α_v_β_5_ may be a biomarker of lymph node metastasis and overall survival in non-small cell lung cancer patients [62]. Integrin α_v_β_5_ was also reported to be highly expressed in both endothelia and human glioma cells and regulate angiogenesis and uncontrolled growth of GBM tumours [63]. However, its biological significance in the context of OV therapy is unknown.

The differential response of ZIKV and ZIKV-LAV entry and replication to Axl or integrin α_v_β_5_ gene knockdown in DBTRG and T98G cells suggests that ZIKV entry into human GBM cells depends primarily on Axl and, to some extent, on integrin α_v_β_5_ expression. A < 20% reduction in Axl surface expression and > 50% reduction in integrin α_v_β_5_ surface expression severely restricted ZIKV-OV entry and replication in DBTRG cells—indicating that receptor expression is the primary determinant of ZIKV-OV infection in these cells. In contrast, an approximate 20% reduction in Axl and integrin α_v_β_5_ surface expression in T98G cells only mildly impacted viral entry and replication, thus highlighting the importance of other host cytoplasmic factors required for viral entry and replication in T98G cells. Although we did not determine the surface expression of Axl and integrin α_v_β_5_ in HUVEC, HBMEC, and terminally differentiated neurons, integrin α_v_ is known to be expressed in majority of cells. The Human Protein Atlas report that Axl and integrin β_5_ are highly expressed in neuroprogenitor cells (NPC) but not in terminally differentiated neurons [64, 65], indicating that the GBM selectivity of ZIKV-LAV infection is partly explained by differential Axl and integrin α_v_β_5_ expression. On the other hand, integrin α_v_β_5_ was reported highly expressed in glioblastoma endothelia [63, 66], while HUVECs exhibit high expression levels of Axl [53]. How the expression of Axl and integrin α_v_β_5_ on endothelia contributes to cancer selectivity of ZIKV-LAV is currently unclear.

The attenuation of natural ZIKV virulence is manifested in reduced DN-1 and DN-2 infectivity in the brain, gonads, and other susceptible tissues [35]. The reduced neurotropism also enhances cancer selectivity of ZIKV-LAV and, consequently, affords wider therapeutic window compared to wild-type ZIKV. This GBM selectivity of ZIKV-LAV infection is its primary advantage over other OVs currently in development for human GBM, which include herpes simplex virus (HSV), adenovirus, measles virus, and poliovirus [14, 67]. HSV is naturally neurotropic and brain infection is the main consequence of GBM therapy with HSV-OV. This required significant virus attenuation to make HSV-OV a viable option for GBM therapy [68]. Another advantage of ZIKV-OV is the absence of host natural immunity to ZIKV, which is a significant challenge for OVs derived from pathogenic viruses for which humans have been widely immunized by either vaccines (*e.g.* poliovirus and measles virus) or continuous lifelong exposure (*e.g.* adenovirus).

**Fig. 7:**
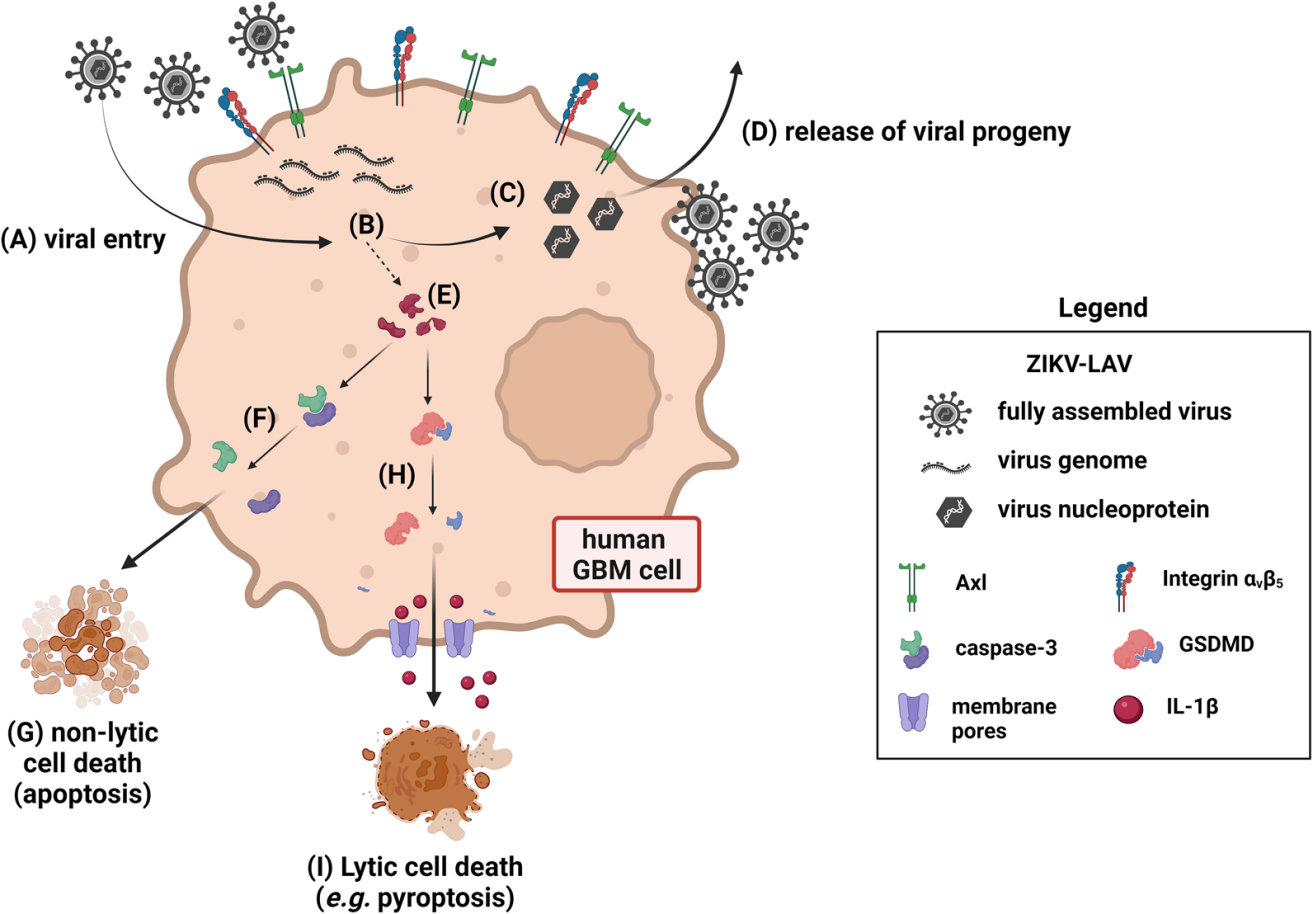
Proposed model of human GBM cell death mediated by ZIKV-LAV infection. **A**–**D** General virus infection life cycle. **A** ZIKV-LAV enters human GBM cells through Axl and integrin α_v_β_5_ cellular receptors. **B** The ZIKV-LAV genome is translated to express viral proteins and the viral genome is replicated. **C** The viral genome and viral proteins assemble the nucleoprotein in preparation for (**D**) release of progeny into the surroundings with concomitant viral incorporation of host cell membrane. **E** Cellular and viral proteins expressed during ZIKV-LAV infection are also responsible for cell death in human GBM. **F**–**G** cleavage of caspase-3 leads to non-lytic or non-inflammatory cell death by apoptosis; **H**–**I** cleavage of gasdermin-D (GSDMD) leads to the formation of membrane pore complexes that shuttles inflammatory IL-1β outside of the cells. GSDMD cleavage also leads to inflammasome activation and lytic and inflammatory cell death by pyroptosis. Image created with BioRender.com

In summary, we propose a model for oncolytic targeting of human GBM based on our observations of ZIKV-LAV infection of human GBM cells and selective killing of these cells (Fig. 7). ZIKV-LAV strains, as well as the parent ZIKV HPF, enter human GBM cells through either Axl, integrin α_v_β_5_ or both receptors expressed on the cell surface. Once inside the cells, the virus replicates its genome and expresses viral proteins, including the envelope (Env) protein. Virus progenies are subsequently assembled and released into the extracellular environment to infect more GBM cells. Inside the cells, viral proteins and virus-induced cellular proteins initiate a molecular cascade that ultimately leads to cancer cell death. This could be either through apoptotic pathways that involve caspase-3 cleavage or through or lytic cell death pathways such as pyroptosis that involves the cleavage of gasdermin-D and subsequent release of IL-1β pro-inflammatory cytokine from infected cells. Though a long way down the road, ZIKV-LAV-mediated cancer cell death could be developed as a promising therapeutic option against deadly GBM.

## Materials and methods

### Cells, viruses, and culture conditions

Human GBM cell lines DBTRG (CRL-2020) and T98G (CRL-1690), as well as monkey kidney Vero (CCL-81) cells were obtained from American Type Culture Collection (ATCC). These cells were cultured in Dulbecco’s Modified Eagle’s Medium (DMEM) supplemented with FBS (10% ^v^/_v_). Primary human umbilical vein endothelial cells (HUVEC) were purchased from Lonza (cat. no. C2591A) and cultured in EGM^™^-2 (Endothelial cell Growth Mmedium-2) BulletKit^™^ (cat. no. CC-3162), while human brain microvascular endothelial cells (HBMEC) were purchased from Cell Systems (cat. no. ACBRI 376) and cultured in Complete serum-free media kit from RocketFuel^™^ (cat. no. SF-4Z0-500). Terminally-differentiated human neurons derived from iPSCs were generated as described previously [69]. These cells were cultured in AGM^™^ (Astrocyte Growth Medium) BulletKit^™^ (Lonza CC-3186). All cells were incubated at 37 °C, 5% CO_2_.

The Zika virus (ZIKV) clinical strain H/PF/2013 isolated from French Polynesia was a generous gift from Prof. Subhash Vasudevan (Programme in Emerging Infectious Diseases, Duke-NUS Medical School). The ZIKV live-attenuated vaccine (ZIKV-LAV) strains DN-1 and DN-2 were previously described elsewhere [35]. All virus stocks used in this experiment were produced by 1 passage in Vero cells cultured in virus growth medium (Dulbecco’s Modified Eagle’s Medium (DMEM) supplemented with FBS (2% ^v^/_v_)).

For infectivity assays in human GBM cells, cells seeded overnight (5 × 10^4^ cells/ well) in 24-well plates were inoculated with either parent ZIKV or ZIKV-LAV strain at 1 MOI (1 plaque-forming unit; pfu / cell) at 37 °C for 90 min. Viral cultures were subsequently incubated in virus growth medium. Infectivity assays in HUVEC and HBMEC were done using EGM^™^-2 BulletKit^™^ and Complete serum-free media kit from RocketFuel^™^, respectively. Infectivity assays in terminally-differentiated human neurons were done using AGM^™^ BulletKit^™^.

### Assessments of cellular infection

Live-cell imaging was performed using Incucyte Zoom^®^ (Sartorius, Michigan, USA). Images were taken every 24 h for 7 days post-infection.

Cells for immunofluorescence (I.F.) staining were seeded overnight in black-walled 24-well *μ*-plates (Ibidi, Munich, Germany) and subsequently inoculated with either parent ZIKV or ZIKV-LAV strain at 1 MOI. At 48 h post-infection, cells were processed for I.F. staining as described previously [70]. Neurons were detected with antibody against microtubule-associated protein 2 (MAP2) (cat. no. AB5392; Abcam, Singapore), while infected cells were detected with 4G2 monoclonal antibody against flavivirus envelope (Env) protein (cat. no. NBP2-52709; Novus Biologicals, Colorado, USA). Images were captured using Olympus Ix83 microscope and Olympus CellSens^®^ Dimension software (Olympus, Tokyo, Japan). All phase-contrast and fluorescence images were processed using ImageJ software (National Institutes of Health, USA).

Cell viability assays were performed by fixing the virus-inoculated cells (seeded in 24-well plates) in 4% paraformaldehyde (PFA) overnight at room temp. Fixed cells were stained with 1% crystal violet in 95% ethanol and dried overnight. Crystal violet inside the cells was extracted by shaking them with 2% SDS in PBS for 1 h at room temp. The amount of crystal violet extracted from the cells was detected using fluorescence plate reader at 570 nm (Tecan M200, Männendorf, Switzerland).

### Determination of viral titre

Viral titres were determined by extracting viral RNA from culture supernatants using QIAamp^®^ viral RNA mini kit (Qiagen, Hilden, Germany). Similarly, the cellular replication of virus was assessed by extracting total cellular RNA using Qiagen RNEasy mini kit. Absolute quantitation of viral RNA was performed by qPCR with a standard curve as described previously [71].

### Clonogenicity assays

Cells seeded cells in 6-well plates (1 × 10^5^ cell/ well) overnight were inoculated with either parent ZIKV or ZIKV-LAV strain at 1 MOI. On days 2 and 5 post-inoculation, cells were trypsinized and reseeded into 24-well plates at a low cell density (5,000 cells / well). Plates were incubated for 7 days, fixed in 4% PFA overnight, and stained with 1% crystal violet in 95% ethanol. After drying overnight, crystal violet was extracted with 2% SDS in PBS and detected using fluorescence plate reader.

### Detection of apoptosis and lytic cell death

Apoptosis and lysis in infected cells were determined by various methods, including luminescence ELISA-based assays, flow cytometry, and Western blot.

Caspase 3/7 cleavage in infected cells was measured with caspase 3/7-Glo luminescence assay kit (Promega, Wisconsin, USA), following the manufacturer’s protocol. Similarly, IL-1β release into the culture supernatant was determined using Lumit^™^ human IL-1β immunoassay kit (Promega, Wisconsin, USA), and lactate dehydrogenase release following lytic cell death was detected using LDH-Glo^™^ cytotoxicity assay kit (Promega, Wisconsin, USA), following the manufacturer’s protocol.

Western blot assay was used to detect cleaved caspase-3 (cat. no. 9661 s; Cell Signaling Technology, Massachusetts, USA), full-length caspase-3 (cat. no. 9662 s; Cell Signaling Technology), and gasdermin-D (GSDMD; cat. no. ab155233; Abcam). Total protein (100 μg per cell lysate) was resolved in 15% SDS-PAGE gel and transferred onto PVDF membrane. The membranes were blocked with 5% skimmed milk prior to probing with different antibodies.

Apoptotic and necrotic cells were simultaneously detected using apoptosis/ necrosis assay kit (Abcam, Singapore), following the manufacturer’s protocol. Briefly, human GBM cells (5 × 10^4^ cells/ well) seeded in 24-well plates overnight were inoculated with virus (need to mention specific strain?) at 1 MOI. At the time of harvesting, cells were gently trypsinized (0.25% trypsin, 25 mM EDTA) and resuspended in assay buffer provided in the kit. The cells were simultaneously stained with Apopxin, 7-AAD, and CytoCalcein Violet 450 by incubating them with the dyes for 30 min at 4 °C in the dark and washing twice with the assay buffer. Labelled cell populations were detected by flow cytometry using MACSQuant VYB (Miltenyi Biotec, North Rhine-Westphalia, Germany), and data were analyzed using FlowJo V10.8.0 (BD Biosciences, USA). Apopxin (λ_Ex_ = 490 nm; λ_Em_ = 525 nm) was excited by a 488 nm laser and detected using the V2 detector (λ_Em_ = 525; band-pass = 50 nm); 7-AAD (λ_Ex_ = 546 nm; λ_Em_ = 647 nm) was excited using a561 nm laser and detected using the Y3 detector (λ_Em_ = 661; band-pass = 20 nm); and CytoCalcein Violet 450 (λ_Ex_ = 405 nm; λ_Em_ = 450 nm) was excited using a 405 nm laser and detected using the V1 detector (λ_Em_ = 452; band-pass = 45 nm).

### Modulation of Axl and integrin α_v_β_5_ cellular expression

The expression of *AXL* (Axl), *ITGAV* (integrin α_v_), and *ITGB5* (integrin β_5_) genes in human GBM cells was determined by relative qPCR using Luna^®^ Universal qPCR Master Mix (New England Biolabs, Massachusetts, USA) and the following primer pairs: hAXL-F (5'-CCG TGG ACC TAC TCT GGC T -3') and h-AXL-R (5'- CCT TGG CGT TAT GGG CTT C-3'); h-ITGAV-F (5'- ATC TGT GAG GTC GAA ACA GGA-3') and h-ITGAV-R (5'-TGG AGC ATA CTC AAC AGT CTT TG-3'); h-ITGB5-F (5'-TCT CGG TGT GAT CTG AGG G-3') and h-ITGB5-R (5'-TGG CGA ACC TGT AGC TGG A-3').. Human GBM cells (1 × 10^5^ cells / well) seeded in 24-well plates overnight were transfected with siRNAs (Integrated DNA Technologies, Iowa, USA) using Lipofectamine RNAiMAX (Themo Fisher Scientific, Massachusetts, USA) to knockdown the expression of these genes. siRNA sequences are available upon request. Total cellular RNAs were extracted using Qiagen RNEasy mini kit, and gene expression was evaluated using relative qPCR, with the expression of the housekeeping gene *GAPDH* used as control.

The expression of the proteins on the cell surface was evaluated by flow cytometry using fluorophore-tagged antibodies against Axl (BD OptiBuild^™^ BV650 anti-human AXL mAb clone 108724) and integrin α_v_β_5_ (BD Biosciences Alexa Fluor 647 anti-human integrin α_v_β_5_). Briefly, cells were harvested by trypsinization on either day 1 after transfection of anti-AXL siRNA or on day 2 after transfection of anti-integrin α_v_β_5_ siRNA. Cells were blocked in 5% BSA/ PBS for 30 min at 4 °C and subsequently incubated with probing antibodies for 30 min and DAPI for 10 min at 4 °C. Antibody-labelled cells were identified by Flow Cytometry (Fortessa, BD Biosciences, USA) and analyzed using FlowJo V10.8.0 (BD Biosciences, USA).

### Virus entry assay

At 48 h after siRNA treatment, human GBM cells were treated with 5 mM sodium azide and 2-deoxyglucose (NaN_3_ + 2DG) for 30 min at 37 °C. Subsequently, cells were inoculated with virus at 1 MOI for 1.5 h at 37 °C. After infection, the remaining viruses attached to cells were digested with pronase (1 mg/ ml) for 30 min at 37 °C. Total cellular RNA was then extracted from the cells using Qiagen RNEasy kit, and viral RNA was quantified with real-time qPCR using ZIKV-specific primers.

### Statistical analysis and data visualization

All data visualization and statistical analyses were performed in Prism v9.0 (GraphPad Software, USA). Means between two groups were compared by nonparametric Mann–Whitney test, while means between > 2 groups were compared by Kruskal–Wallis test with Dunn’s post-hoc correction. Linear regression analyses on scatter plots were performed using Spearman’s coefficient (*ρ*). Results were considered statistically significant at *p* < 0.05.

## Supplementary Information

**Additional file 1: Figure S1.** ZIKV-LAV early infection at day 2 inhibits clonogenic reproduction of human GBM cells. (**A**) Representative clonogenicity of plates of cells inoculated with virus at 1 MOI and subsequently replated at day 2 post-infection. (**B**) Quantification of clonogenicity data, which are presented as mean ± SEM. Non-parametric Kruskal–Wallis test with Dunn’s post-hoc correction was used to compare groups. *p*-values are shown accordingly: *, *p* < 0.05. **, *p* < 0.005, ***, *p* < 0.001. **Figure S2.** ZIKV-LAV does not infect human embryonic vascular endothelial cells (HUVEC) ZIKV-LAV infection of HUVEC cells over 3 days, evaluated by measuring (**A**) changes in cell viability and (**B**) viral copies detected in infected cells. Data are presented as mean ± SEM. Non-parametric Kruskal–Wallis test with Dunn’s post-hoc correction was used to compare groups. **Figure S3.** Confirmation of reduction in gene expression following siRNA-mediated knockdown. Gene expression of (**A**) Axl and (**B**) integrin α_v_β_5_ in human GBM cells infected with ZIKV-LAV strains, following siRNA-mediated expression knockdown. *SCR*, scrambled siRNA. Values are presented as gene expression relative to cells treated with SCR siRNA. Data are presented as mean ± SD. Non-parametric Kruskal–Wallis test with Dunn’s post-hoc correction was used to compare groups. *p*-values are shown accordingly: *, *p* < 0.05. **, *p* < 0.005.

## Data Availability

All data are available upon request.

## References

[1] Alexander BM, Cloughesy TF. Adult glioblastoma. J Clin Oncol. 2017;35(21):2402–9. 10.1200/JCO.2017.73.0119. 28640706 10.1200/JCO.2017.73.0119

[2] Brain GBD, C. N. S. Cancer Collaborators Other. Global, regional, and national burden of brain and other Cns cancer, 1990–2016: a systematic analysis for the global burden of disease study 2016. Lancet Neurol. 2019;18(4):376–93. 10.1016/S1474-4422(18)30468-X. 30797715 10.1016/S1474-4422(18)30468-X PMC6416167

[3] Thakkar JP, Dolecek TA, Horbinski C, Ostrom QT, Lightner DD, Barnholtz-Sloan JS, Villano JL. Epidemiologic and molecular prognostic review of glioblastoma. Cancer Epidemiol Biomarkers Prev. 2014;23(10):1985–96. 10.1158/1055-9965.EPI-14-0275. 25053711 10.1158/1055-9965.EPI-14-0275 PMC4185005

[4] Stupp R, Hegi ME, Mason WP, van den Bent MJ, Taphoorn MJ, Janzer RC, Ludwin SK, Allgeier A, Fisher B, Belanger K, Hau P, Brandes AA, Gijtenbeek J, Marosi C, Vecht CJ, Mokhtari K, Wesseling P, Villa S, Eisenhauer E, Gorlia T, Weller M, Lacombe D, Cairncross JG, Mirimanoff RO, Research European Organisation for, Tumour Treatment of Cancer Brain, Groups Radiation Oncology, and Group National Cancer Institute of Canada Clinical Trials. Effects of radiotherapy with concomitant and adjuvant temozolomide versus radiotherapy alone on survival in glioblastoma in a randomised phase iii study: 5-year analysis of the Eortc-Ncic Trial. Lancet Oncol. 2009;10(5):459–66. 10.1016/S1470-2045(09)70025-7. 19269895 10.1016/S1470-2045(09)70025-7

[5] Feng E, Sui C, Wang T, Sun G. Temozolomide with or without radiotherapy in patients with newly diagnosed glioblastoma multiforme: a meta-analysis. Eur Neurol. 2017;77(3–4):201–10. 10.1159/000455842. 28192785 10.1159/000455842

[6] Chacko AM, Li C, Pryma DA, Brem S, Coukos G, Muzykantov V. Targeted delivery of antibody-based therapeutic and imaging agents to cns tumors: crossing the blood-brain barrier divide. Expert Opin Drug Deliv. 2013;10(7):907–26. 10.1517/17425247.2013.808184. 23751126 10.1517/17425247.2013.808184 PMC4089357

[7] Mikitsh JL, Chacko AM. Pathways for small molecule delivery to the central nervous system across the blood-brain barrier. Perspect Medicin Chem. 2014;6:11–24. 10.4137/PMC.S13384. 24963272 10.4137/PMC.S13384 PMC4064947

[8] Bien-Moller S, Balz E, Herzog S, Plantera L, Vogelgesang S, Weitmann K, Seifert C, Fink MA, Marx S, Bialke A, Venugopal C, Singh SK, Hoffmann W, Rauch BH, Schroeder HWS. Association of glioblastoma multiforme stem cell characteristics, differentiation, and microglia marker genes with patient survival. Stem Cells Int. 2018;2018:9628289. 10.1155/2018/9628289. 29535786 10.1155/2018/9628289 PMC5822829

[9] Yi Y, Hsieh IY, Huang X, Li J, Zhao W. Glioblastoma stem-like cells: characteristics, microenvironment, and therapy. Front Pharmacol. 2016;7:477. 10.3389/fphar.2016.00477. 28003805 10.3389/fphar.2016.00477 PMC5141588

[10] Sampetrean O, Saya H. Characteristics of glioma stem cells. Brain Tumor Pathol. 2013;30(4):209–14. 10.1007/s10014-013-0141-5. 23584571 10.1007/s10014-013-0141-5

[11] Russell SJ, Barber GN. Oncolytic viruses as antigen-agnostic cancer vaccines. Cancer Cell. 2018;33(4):599–605. 10.1016/j.ccell.2018.03.011. 29634947 10.1016/j.ccell.2018.03.011 PMC5918693

[12] Martikainen M, Essand M. Virus-based immunotherapy of glioblastoma. Cancers (Basel). 2019;11(2):186. 10.3390/cancers11020186. 30764570 10.3390/cancers11020186 PMC6407011

[13] Chiocca EA, Nassiri F, Wang J, Peruzzi P, Zadeh G. Viral and other therapies for recurrent glioblastoma: is a 24-month durable response unusual? Neuro Oncol. 2019;21(1):14–25. 10.1093/neuonc/noy170. 30346600 10.1093/neuonc/noy170 PMC6303472

[14] Rius-Rocabert S, Garcia-Romero N, Garcia A, Ayuso-Sacido A, Nistal-Villan E. Oncolytic virotherapy in glioma tumors. Int J Mol Sci. 2020;21(20):7604. 10.3390/ijms21207604. 33066689 10.3390/ijms21207604 PMC7589679

[15] Hamad A, Yusubalieva GM, Baklaushev VP, Chumakov PM, Lipatova AV. Recent developments in glioblastoma therapy: oncolytic viruses and emerging future strategies. Viruses. 2023;15(2):547. 10.3390/v15020547. 36851761 10.3390/v15020547 PMC9958853

[16] Frampton JE. Teserpaturev/G47delta: first approval. BioDrugs. 2022;36(5):667–72. 10.1007/s40259-022-00553-7. 36098872 10.1007/s40259-022-00553-7

[17] Tang H, Hammack C, Ogden SC, Wen Z, Qian X, Li Y, Yao B, Shin J, Zhang F, Lee EM, Christian KM, Didier RA, Jin P, Song H, Ming GL. Zika virus infects human cortical neural progenitors and attenuates their growth. Cell Stem Cell. 2016;18(5):587–90. 10.1016/j.stem.2016.02.016. 26952870 10.1016/j.stem.2016.02.016 PMC5299540

[18] Li C, Xu D, Ye Q, Hong S, Jiang Y, Liu X, Zhang N, Shi L, Qin CF, Xu Z. Zika virus disrupts neural progenitor development and leads to microcephaly in mice. Cell Stem Cell. 2016;19(1):120–6. 10.1016/j.stem.2016.04.017. 27179424 10.1016/j.stem.2016.04.017

[19] Cugola FR, Fernandes IR, Russo FB, Freitas BC, Dias JL, Guimaraes KP, Benazzato C, Almeida N, Pignatari GC, Romero S, Polonio CM, Cunha I, Freitas CL, Brandao WN, Rossato C, Andrade DG, Faria Dde P, Garcez AT, Buchpigel CA, Braconi CT, Mendes E, Sall AA, Zanotto PM, Peron JP, Muotri AR, Beltrao-Braga PC. The Brazilian Zika virus strain causes birth defects in experimental models. Nature. 2016;534(7606):267–71. 10.1038/nature18296. 27279226 10.1038/nature18296 PMC4902174

[20] Aliota MT, Caine EA, Walker EC, Larkin KE, Camacho E, Osorio JE. Characterization of lethal Zika virus infection in Ag129 Mice. PLoS Negl Trop Dis. 2016;10(4):e0004682. 10.1371/journal.pntd.0004682. 27093158 10.1371/journal.pntd.0004682 PMC4836712

[21] Noguchi KK, Swiney BS, Williams SL, Huffman JN, Lucas K, Wang SH, Kapral KM, Li A, Dikranian KT. Zika virus infection in the developing mouse produces dramatically different neuropathology dependent on viral strain. J Neurosci. 2020;40(5):1145–61. 10.1523/JNEUROSCI.1376-19.2019. 31836659 10.1523/JNEUROSCI.1376-19.2019 PMC6988996

[22] Manangeeswaran M, Ireland DD, Verthelyi D. Zika (Prvabc59) infection is associated with T cell infiltration and neurodegeneration in Cns of immunocompetent neonatal C57bl/6 mice. PLoS Pathog. 2016;12(11):e1006004. 10.1371/journal.ppat.1006004. 27855206 10.1371/journal.ppat.1006004 PMC5113993

[23] Victorio CBL, Msallam R, Novera W, Ong J, Yang TJ, Ganasarajah A, Low J, Watanabe S, Chacko AM. Tspo expression in a Zika virus murine infection model as an imaging target for acute infection-induced neuroinflammation. Eur J Nucl Med Mol Imaging. 2023;50(3):742–55. 10.1007/s00259-022-06019-w. 36348095 10.1007/s00259-022-06019-w PMC9852192

[24] Kuszpit K, Hollidge BS, Zeng X, Stafford RG, Daye S, Zhang X, Basuli F, Golden JW, Swenson RE, Smith DR, Bocan TM. [(18)F]Dpa-714 Pet imaging reveals global neuroinflammation in Zika virus-infected mice. Mol Imaging Biol. 2018;20(2):275–83. 10.1007/s11307-017-1118-2. 28900831 10.1007/s11307-017-1118-2 PMC5862915

[25] Zhu Z, Gorman MJ, McKenzie LD, Chai JN, Hubert CG, Prager BC, Fernandez E, Richner JM, Zhang R, Shan C, Tycksen E, Wang X, Shi PY, Diamond MS, Rich JN, Chheda MG. Zika virus has oncolytic activity against glioblastoma stem cells. J Exp Med. 2017;214(10):2843–57. 10.1084/jem.20171093. 28874392 10.1084/jem.20171093 PMC5626408

[26] Kaid C, Goulart E, Caires-Junior LC, Araujo BHS, Soares-Schanoski A, Bueno HMS, Telles-Silva KA, Astray RM, Assoni AF, Junior AFR, Ventini DC, Puglia ALP, Gomes RP, Zatz M, Okamoto OK. Zika virus selectively kills aggressive human embryonal CNS tumor cells in vitro and in vivo. Cancer Res. 2018;78(12):3363–74. 10.1158/0008-5472.CAN-17-3201. 29700002 10.1158/0008-5472.CAN-17-3201

[27] Lubin JA, Zhang RR, Kuo JS. Zika virus has oncolytic activity against glioblastoma stem cells. Neurosurgery. 2018;82(5):E113–4. 10.1093/neuros/nyy047. 29669124 10.1093/neuros/nyy047 PMC6257021

[28] Crane AT, Chrostek MR, Krishna VD, Shiao M, Toman NG, Pearce CM, Tran SK, Sipe CJ, Guo W, Voth JP, Vaid S, Xie H, Lu WC, Swanson W, Grande AW, Schleiss MR, Bierle CJ, Cheeran MC, Low WC. Zika virus-based immunotherapy enhances long-term survival of rodents with brain tumors through upregulation of memory T-cells. PLoS ONE. 2020;15(10):e0232858. 10.1371/journal.pone.0232858. 33002018 10.1371/journal.pone.0232858 PMC7529292

[29] Nair S, Mazzoccoli L, Jash A, Govero J, Bais SS, Hu T, Fontes-Garfias CR, Shan C, Okada H, Shresta S, Rich JN, Shi PY, Diamond MS, Chheda MG. Zika virus oncolytic activity requires Cd8+ T cells and is boosted by immune checkpoint blockade. JCI Insight. 2021. 10.1172/jci.insight.144619. 33232299 10.1172/jci.insight.144619 PMC7821591

[30] Trus I, Berube N, Jiang P, Rak J, Gerdts V, Karniychuk U. Zika virus with increased Cpg dinucleotide frequencies shows oncolytic activity in glioblastoma stem cells. Viruses. 2020;12(5):579. 10.3390/v12050579. 32466170 10.3390/v12050579 PMC7290362

[31] Chen Q, Wu J, Ye Q, Ma F, Zhu Q, Wu Y, Shan C, Xie X, Li D, Zhan X, Li C, Li XF, Qin X, Zhao T, Wu H, Shi PY, Man J, Qin CF. Treatment of human glioblastoma with a live attenuated Zika virus vaccine candidate. mBio. 2018. 10.1128/mBio.01683-18. 30228241 10.1128/mBio.01683-18 PMC6143740

[32] Kaid C, Rads Madi R, Astray E, Goulart LC, Caires-Junior TG, Mitsugi ACR, Moreno MF, Castro-Amarante LR, Pereira Bfmm Porchia, de Andrade TO, Landini V, Sanches DS, Pires CG, Tanioka RKO, Pereira MCL, Barbosa IN, Massoco CO, Ferreira LCS, Okamoto OK, Zatz M. Safety, tumor reduction, and clinical impact of Zika virus injection in dogs with advanced-stage brain tumors. Mol Ther. 2020;28(5):1276–86. 10.1016/j.ymthe.2020.03.004. 32220305 10.1016/j.ymthe.2020.03.004 PMC7210722

[33] Shan C, Muruato AE, Nunes BTD, Luo H, Xie X, Medeiros DBA, Wakamiya M, Tesh RB, Barrett AD, Wang T, Weaver SC, Vasconcelos PFC, Rossi SL, Shi PY. A live-attenuated Zika virus vaccine candidate induces sterilizing immunity in mouse models. Nat Med. 2017;23(6):763–7. 10.1038/nm.4322. 28394328 10.1038/nm.4322 PMC6276361

[34] Udenze D, Trus I, Berube N, Karniychuk U. Cpg content in the Zika virus genome affects infection phenotypes in the adult brain and fetal lymph nodes. Front Immunol. 2022;13:943481. 10.3389/fimmu.2022.943481. 35983032 10.3389/fimmu.2022.943481 PMC9379343

[35] Kwek SS, Watanabe S, Chan KR, Ong EZ, Tan HC, Ng WC, Nguyen MTX, Gan ES, Zhang SL, Chan KWK, Tan JH, Sessions OM, Manuel M, Pompon J, Chua C, Hazirah S, Tryggvason K, Vasudevan SG, Ooi EE. A systematic approach to the development of a safe live attenuated Zika vaccine. Nat Commun. 2018;9(1):1031. 10.1038/s41467-018-03337-2. 29531213 10.1038/s41467-018-03337-2 PMC5847552

[36] Dirkse A, Golebiewska A, Buder T, Nazarov PV, Muller A, Poovathingal S, Brons NHC, Leite S, Sauvageot N, Sarkisjan D, Seyfrid M, Fritah S, Stieber D, Michelucci A, Hertel F, Herold-Mende C, Azuaje F, Skupin A, Bjerkvig R, Deutsch A, Voss-Bohme A, Niclou SP. Stem cell-associated heterogeneity in glioblastoma results from intrinsic tumor plasticity shaped by the microenvironment. Nat Commun. 2019;10(1):1787. 10.1038/s41467-019-09853-z. 30992437 10.1038/s41467-019-09853-z PMC6467886

[37] Lauko A, Lo A, Ahluwalia MS, Lathia JD. Cancer cell heterogeneity & plasticity in glioblastoma and brain tumors. Semin Cancer Biol. 2022;82:162–75. 10.1016/j.semcancer.2021.02.014. 33640445 10.1016/j.semcancer.2021.02.014 PMC9618157

[38] Barrows NJ, Campos RK, Liao KC, Prasanth KR, Soto-Acosta R, Yeh SC, Schott-Lerner G, Pompon J, Sessions OM, Bradrick SS, Garcia-Blanco MA. Biochemistry and molecular biology of flaviviruses. Chem Rev. 2018;118(8):4448–82. 10.1021/acs.chemrev.7b00719. 29652486 10.1021/acs.chemrev.7b00719 PMC5937540

[39] Valadao AL, Aguiar RS, de Arruda LB. Interplay between inflammation and cellular stress triggered by flaviviridae viruses. Front Microbiol. 2016;7:1233. 10.3389/fmicb.2016.01233. 27610098 10.3389/fmicb.2016.01233 PMC4996823

[40] Miorin L, Maestre AM, Fernandez-Sesma A, Garcia-Sastre A. Antagonism of type i interferon by flaviviruses. Biochem Biophys Res Commun. 2017;492(4):587–96. 10.1016/j.bbrc.2017.05.146. 28576494 10.1016/j.bbrc.2017.05.146 PMC5626595

[41] Anfasa F, Goeijenbier M, Widagdo W, Siegers JY, Mumtaz N, Okba N, van Riel D, Rockx B, Koopmans MPG, Meijers JCM, Martina BEE. Zika virus infection induces elevation of tissue factor production and apoptosis on human umbilical vein endothelial cells. Front Microbiol. 2019;10:817. 10.3389/fmicb.2019.00817. 31068911 10.3389/fmicb.2019.00817 PMC6491739

[42] Peng H, Liu B, Yves TD, He Y, Wang S, Tang H, Ren H, Zhao P, Qi Z, Qin Z. Zika virus induces autophagy in human umbilical vein endothelial cells. Viruses. 2018;10(5):259. 10.3390/v10050259. 29762492 10.3390/v10050259 PMC5977252

[43] Mladinich MC, Schwedes J, Mackow ER. Zika virus persistently infects and is basolaterally released from primary human brain microvascular endothelial cells. mBio. 2017. 10.1128/mBio.00952-17. 28698279 10.1128/mBio.00952-17 PMC5513708

[44] Papa MP, Meuren LM, Coelho SVA, Lucas CGO, Mustafa YM, Lemos Matassoli F, Silveira PP, Frost PS, Pezzuto P, Ribeiro MR, Tanuri A, Nogueira ML, Campanati L, Bozza MT, Paula Neto HA, Pimentel-Coelho PM, Figueiredo CP, de Aguiar RS, de Arruda LB. Zika virus infects, activates, and crosses brain microvascular endothelial cells, without barrier disruption. Front Microbiol. 2017;8:2557. 10.3389/fmicb.2017.02557. 29312238 10.3389/fmicb.2017.02557 PMC5743735

[45] Kaufman HL, Kohlhapp FJ, Zloza A. Oncolytic viruses: a new class of immunotherapy drugs. Nat Rev Drug Discov. 2015;14(9):642–62. 10.1038/nrd4663. 26323545 10.1038/nrd4663 PMC7097180

[46] Zhao Z, Li Q, Ashraf U, Yang M, Zhu W, Gu J, Chen Z, Gu C, Si Y, Cao S, Ye J. Zika virus causes placental pyroptosis and associated adverse fetal outcomes by activating Gsdme. Elife. 2022. 10.7554/eLife.73792. 35972780 10.7554/eLife.73792 PMC9381041

[47] Wen C, Yu Y, Gao C, Qi X, Cardona CJ, Xing Z. Concomitant pyroptotic and apoptotic cell death triggered in macrophages infected by Zika virus. PLoS ONE. 2022;17(4):e0257408. 10.1371/journal.pone.0257408. 35446851 10.1371/journal.pone.0257408 PMC9022797

[48] Wen C, Yu Y, Gao C, Qi X, Cardona CJ, Xing Z. Ripk3-dependent necroptosis is induced and restricts viral replication in human astrocytes infected with Zika virus. Front Cell Infect Microbiol. 2021;11:637710. 10.3389/fcimb.2021.637710. 33796483 10.3389/fcimb.2021.637710 PMC8007970

[49] Ma J, Ramachandran M, Jin C, Quijano-Rubio C, Martikainen M, Yu D, Essand M. Characterization of virus-mediated immunogenic cancer cell death and the consequences for oncolytic virus-based immunotherapy of cancer. Cell Death Dis. 2020;11(1):48. 10.1038/s41419-020-2236-3. 31969562 10.1038/s41419-020-2236-3 PMC6976683

[50] Yau C, Gan ES, Kwek SS, Tan HC, Ong EZ, Hamis NZ, Rivino L, Chan KR, Watanabe S, Vasudevan SG, Ooi EE. Live vaccine infection burden elicits adaptive humoral and cellular immunity required to prevent Zika virus infection. EBioMedicine. 2020;61:103028. 10.1016/j.ebiom.2020.103028. 33045466 10.1016/j.ebiom.2020.103028 PMC7553235

[51] Chen L, Zhou C, Chen Q, Shang J, Liu Z, Guo Y, Li C, Wang H, Ye Q, Li X, Zu S, Li F, Xia Q, Zhou T, Li A, Wang C, Chen Y, Wu A, Qin C, Man J. Oncolytic Zika virus promotes intratumoral T cell infiltration and improves immunotherapy efficacy in glioblastoma. Mol Ther Oncolytics. 2022;24:522–34. 10.1016/j.omto.2022.01.011. 35229030 10.1016/j.omto.2022.01.011 PMC8851082

[52] Uhlen M, Fagerberg L, Hallstrom BM, Lindskog C, Oksvold P, Mardinoglu A, Sivertsson A, Kampf C, Sjostedt E, Asplund A, Olsson I, Edlund K, Lundberg E, Navani S, Szigyarto CA, Odeberg J, Djureinovic D, Takanen JO, Hober S, Alm T, Edqvist PH, Berling H, Tegel H, Mulder J, Rockberg J, Nilsson P, Schwenk JM, Hamsten M, von Feilitzen K, Forsberg M, Persson L, Johansson F, Zwahlen M, von Heijne G, Nielsen J, Ponten F. Proteomics. Tissue-based map of the human proteome. Science. 2015;347(6220):1260419. 10.1126/science.1260419. 25613900 10.1126/science.1260419

[53] Richard AS, Shim BS, Kwon YC, Zhang R, Otsuka Y, Schmitt K, Berri F, Diamond MS, Choe H. Axl-dependent infection of human fetal endothelial cells distinguishes Zika virus from other pathogenic flaviviruses. Proc Natl Acad Sci U S A. 2017;114(8):2024–9. 10.1073/pnas.1620558114. 28167751 10.1073/pnas.1620558114 PMC5338370

[54] Liu S, DeLalio LJ, Isakson BE, Wang TT. Axl-mediated productive infection of human endothelial cells by Zika virus. Circ Res. 2016;119(11):1183–9. 10.1161/CIRCRESAHA.116.309866. 27650556 10.1161/CIRCRESAHA.116.309866 PMC5215127

[55] Meertens L, Labeau A, Dejarnac O, Cipriani S, Sinigaglia L, Bonnet-Madin L, Le Charpentier T, Hafirassou ML, Zamborlini A, Cao-Lormeau VM, Coulpier M, Misse D, Jouvenet N, Tabibiazar R, Gressens P, Schwartz O, Amara A. Axl mediates Zika virus entry in human glial cells and modulates innate immune responses. Cell Rep. 2017;18(2):324–33. 10.1016/j.celrep.2016.12.045. 28076778 10.1016/j.celrep.2016.12.045

[56] Li J, Akbani R, Zhao W, Lu Y, Weinstein JN, Mills GB, Liang H. Explore, visualize, and analyze functional cancer proteomic data using the cancer proteome atlas. Cancer Res. 2017;77(21):e51–4. 10.1158/0008-5472.CAN-17-0369. 29092939 10.1158/0008-5472.CAN-17-0369 PMC5679242

[57] Li J, Lu Y, Akbani R, Ju Z, Roebuck PL, Liu W, Yang JY, Broom BM, Verhaak RG, Kane DW, Wakefield C, Weinstein JN, Mills GB, Liang H. Tcpa: a resource for cancer functional proteomics data. Nat Methods. 2013;10(11):1046–7. 10.1038/nmeth.2650. 24037243 10.1038/nmeth.2650 PMC4076789

[58] Paccez JD, Vogelsang M, Parker MI, Zerbini LF. The receptor tyrosine kinase Axl in cancer: biological functions and therapeutic implications. Int J Cancer. 2014;134(5):1024–33. 10.1002/ijc.28246. 23649974 10.1002/ijc.28246

[59] Gay CM, Balaji K, Byers LA. Giving Axl the axe: targeting Axl in human malignancy. Br J Cancer. 2017;116(4):415–23. 10.1038/bjc.2016.428. 28072762 10.1038/bjc.2016.428 PMC5318970

[60] Zhu Z, Mesci P, Bernatchez JA, Gimple RC, Wang X, Schafer ST, Wettersten HI, Beck S, Clark AE, Wu Q, Prager BC, Kim LJY, Dhanwani R, Sharma S, Garancher A, Weis SM, Mack SC, Negraes PD, Trujillo CA, Penalva LO, Feng J, Lan Z, Zhang R, Wessel AW, Dhawan S, Diamond MS, Chen CC, Wechsler-Reya RJ, Gage FH, Hu H, Siqueira-Neto JL, Muotri AR, Cheresh DA, Rich JN. Zika virus targets glioblastoma stem cells through a Sox2-integrin alpha(V)beta(5) axis. Cell Stem Cell. 2020;26(2):187–204. 10.1016/j.stem.2019.11.016. 31956038 10.1016/j.stem.2019.11.016 PMC9628766

[61] Wang S, Zhang Q, Tiwari SK, Lichinchi G, Yau EH, Hui H, Li W, Furnari F, Rana TM. Integrin alphavbeta5 internalizes Zika virus during neural stem cells infection and provides a promising target for antiviral therapy. Cell Rep. 2020;30(4):969–83. 10.1016/j.celrep.2019.11.020. 31956073 10.1016/j.celrep.2019.11.020 PMC7293422

[62] Bai SY, Xu N, Chen C, Song YL, Hu J, Bai CX. Integrin alphavbeta5 as a biomarker for the assessment of non-small cell lung cancer metastasis and overall survival. Clin Respir J. 2015;9(4):457–67. 10.1111/crj.12163. 24815623 10.1111/crj.12163

[63] Roth P, Silginer M, Goodman SL, Hasenbach K, Thies S, Maurer G, Schraml P, Tabatabai G, Moch H, Tritschler I, Weller M. Integrin control of the transforming growth factor-beta pathway in glioblastoma. Brain. 2013;136(Pt 2):564–76. 10.1093/brain/aws351. 23378223 10.1093/brain/aws351

[64] Integrin Β5. The human protein atlas, https://www.proteinatlas.org/ENSG00000082781-ITGB5/single+cell+type. Accessed 15 Jan.

[65] Axl. The Human Protein Atlas, https://www.proteinatlas.org/ENSG00000167601-AXL/single+cell+type. Accessed 15 Jan.

[66] Schittenhelm J, Klein A, Tatagiba MS, Meyermann R, Fend F, Goodman SL, Sipos B. Comparing the expression of integrins alphavbeta3, alphavbeta5, alphavbeta6, alphavbeta8, fibronectin and fibrinogen in human brain metastases and their corresponding primary tumors. Int J Clin Exp Pathol. 2013;6(12):2719–32. 24294359 PMC3843253

[67] Lu VM, Shah AH, Vallejo FA, Eichberg DG, Luther EM, Shah SS, Komotar RJ, Ivan ME. Clinical trials using oncolytic viral therapy to treat adult glioblastoma: a progress report. Neurosurg Focus. 2021;50(2):E3. 10.3171/2020.11.FOCUS20860. 33524946 10.3171/2020.11.FOCUS20860

[68] Kardani K, Sanchez Gil J, Rabkin SD. Oncolytic herpes simplex viruses for the treatment of glioma and targeting glioblastoma stem-like cells. Front Cell Infect Microbiol. 2023;13:1206111. 10.3389/fcimb.2023.1206111. 37325516 10.3389/fcimb.2023.1206111 PMC10264819

[69] Sun AX, Yuan Q, Tan S, Xiao Y, Wang D, Khoo AT, Sani L, Tran HD, Kim P, Chiew YS, Lee KJ, Yen YC, Ng HH, Lim B, Je HS. Direct induction and functional maturation of forebrain gabaergic neurons from human pluripotent stem cells. Cell Rep. 2016;16(7):1942–53. 10.1016/j.celrep.2016.07.035. 27498872 10.1016/j.celrep.2016.07.035

[70] Victorio CBL, Novera W, Tham JY, Watanabe S, Vasudevan SG, Chacko AM. Peptide-conjugated phosphorodiamidate morpholino oligomers for in situ live-cell molecular imaging of dengue virus replication. Int J Mol Sci. 2020;21(23):9260. 10.3390/ijms21239260. 33291644 10.3390/ijms21239260 PMC7730579

[71] Watanabe S, Tan NWW, Chan KWK, Vasudevan SG. Dengue virus and Zika virus serological cross-reactivity and their impact on pathogenesis in mice. J Infect Dis. 2019;219(2):223–33. 10.1093/infdis/jiy482. 30085051 10.1093/infdis/jiy482

