# Supplementary material for: Repurposing of Zika virus live-attenuated vaccine (ZIKV-LAV) strains as oncolytic viruses targeting human glioblastoma multiforme cells
Source: J Transl Med. 2024 Feb 2;22:126. doi: 10.1186/s12967-024-04930-4 (PMC10835997; doi:10.1186/s12967-024-04930-4)
Supplement: Supplementary file 1 — Additional file 1: Figure S1. ZIKV-LAV early infection at day 2 inhibits clonogenic reproduction of human GBM cells. (A) Representative clonogenicity of plates of cells inoculated with virus at 1 MOI and subsequently replated at day 2 post-infection. (B) Quantification of clonogenicity data, which are presented as mean ± SEM. Non-parametric Kruskal–Wallis test with Dunn’s post-hoc correction was used to compare groups. p-values are shown accordingly: *, p < 0.05. **, p < 0.005, ***, p < 0.001. Figure S2. ZIKV-LAV does not infect human embryonic vascular endothelial cells (HUVEC) ZIKV-LAV infection of HUVEC cells over 3 days, evaluated by measuring (A) changes in cell viability and (B) viral copies detected in infected cells. Data are presented as mean ± SEM. Non-parametric Kruskal–Wallis test with Dunn’s post-hoc correction was used to compare groups. Figure S3. Confirmation of reduction in gene expression following siRNA-mediated knockdown. Gene expression of (A) Axl and (B) integrin αvβ5 in human GBM cells infected with ZIKV-LAV strains, following siRNA-mediated expression knockdown. SCR, scrambled siRNA. Values are presented as gene expression relative to cells treated with SCR siRNA. Data are presented as mean ± SD. Non-parametric Kruskal–Wallis test with Dunn’s post-hoc correction was used to compare groups. p-values are shown accordingly: *, p < 0.05. **, p < 0.005. [file 12967_2024_4930_MOESM1_ESM.docx]

**Figure S1**

**
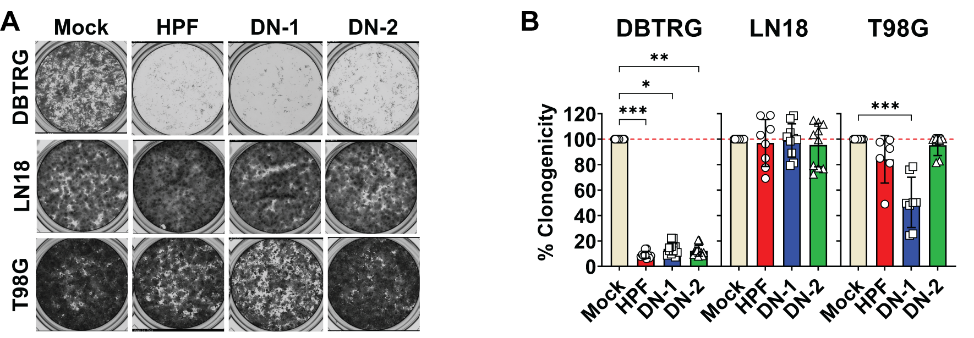
**

**Fig. S1.** *ZIKV-LAV early infection at day 2 inhibits clonogenic reproduction of human GBM cells.* (**A**) Representative clonogenicity of plates of cells inoculated with virus at 1 MOI and subsequently replated at day 2 post-infection. (**B**) Quantification of clonogenicity data, which are presented as mean ± SEM. Non-parametric Kruskal-Wallis test with Dunn’s post-hoc correction was used to compare groups. *p*-values are shown accordingly: *, *p* < 0.05. **, *p* < 0.005, ***, *p* <0.001.

**Figure S2**

**
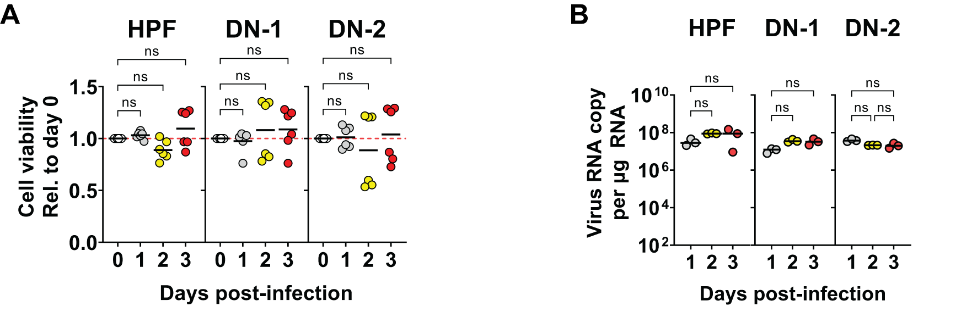
**

**Fig. S2.** *ZIKV-LAV does not infect human embryonic vascular endothelial cells (HUVEC)* ZIKV-LAV infection of HUVEC cells over 3 days, evaluated by measuring (**A**) changes in cell viability and (**B**) viral copies detected in infected cells. Data are presented as mean ± SEM. Non-parametric Kruskal-Wallis test with Dunn’s post-hoc correction was used to compare groups.

**Figure S3**

**
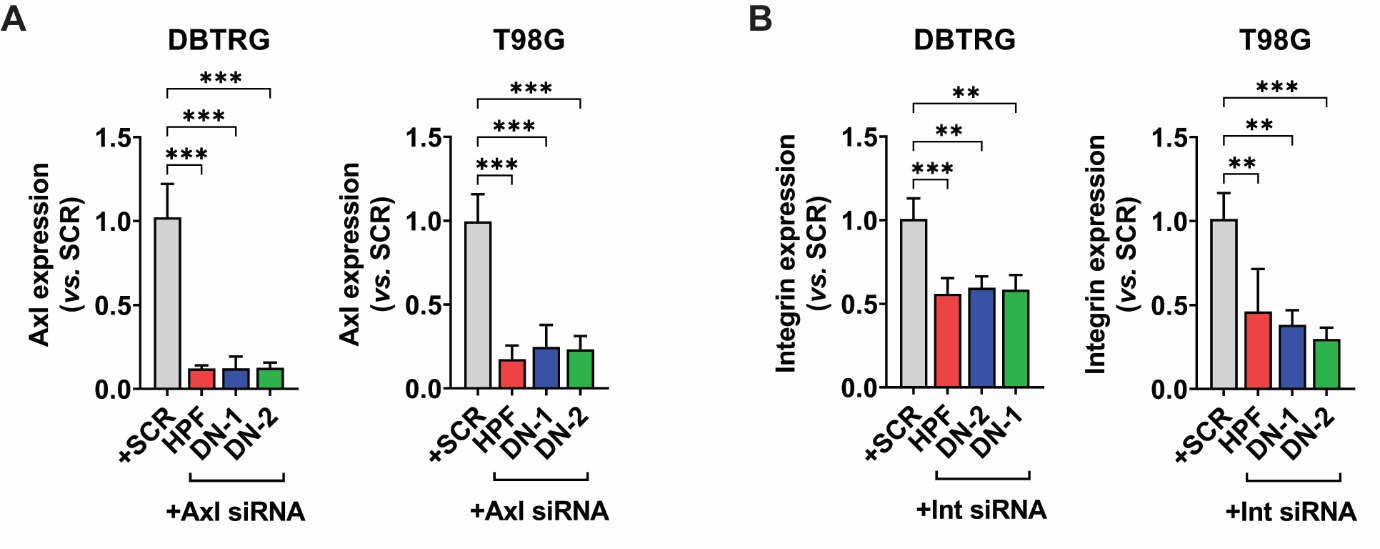
**

**Fig. S3.** *Confirmation of reduction in gene expression following siRNA-mediated knockdown.* Gene expression of (**A**) Axl and (**B**) integrin α_v_β_5_ in human GBM cells infected with ZIKV-LAV strains, following siRNA-mediated expression knockdown. *SCR*, scrambled siRNA. Values are presented as gene expression relative to cells treated with SCR siRNA. Data are presented as mean ± SD. Non-parametric Kruskal-Wallis test with Dunn’s post-hoc correction was used to compare groups. *p*-values are shown accordingly: *, *p* < 0.05. **, *p* < 0.005.
